# Treatable inborn errors of metabolism presenting as cerebral palsy mimics: systematic literature review

**DOI:** 10.1186/s13023-014-0197-2

**Published:** 2014-11-30

**Authors:** Emma L Leach, Michael Shevell, Kristin Bowden, Sylvia Stockler-Ipsiroglu, Clara DM van Karnebeek

**Affiliations:** Provincial Medical Genetics Program, BC Women’s Hospital, Vancouver, Canada; Division of Biochemical Diseases, Department of Pediatrics, BC Children’s Hospital, University of British Columbia, Vancouver, Canada; Department of Pediatrics, Montreal Children’s Hospital, McGill University, Montreal, Canada; Department of Neurology/Neurosurgery, Montreal Children’s Hospital, McGill University, Montreal, Canada; Division of Biochemical Diseases, Department of Pediatrics, BC Children’s Hospital, Vancouver, Canada; Treatable Intellectual Disability Endeavour in British Columbia, Vancouver, Canada; Centre for Molecular Medicine and Therapeutics; Child and Family Research Institute, Vancouver, Canada; Division of Biochemical Diseases, Department of Pediatrics, University of British Columbia, Rm K3-201, 4480 Oak Street, Vancouver, BC V6H 3V4 Canada

**Keywords:** Inherited metabolic diseases, Therapy, Diagnosis, Atypical cerebral palsy, Movement disorders

## Abstract

**Background:**

Inborn errors of metabolism (IEMs) have been anecdotally reported in the literature as presenting with features of cerebral palsy (CP) or misdiagnosed as ‘atypical CP’. A significant proportion is amenable to treatment either directly targeting the underlying pathophysiology (often with improvement of symptoms) or with the potential to halt disease progression and prevent/minimize further damage.

**Methods:**

We performed a systematic literature review to identify all reports of IEMs presenting with CP-like symptoms before 5 years of age, and selected those for which evidence for effective treatment exists.

**Results:**

We identified 54 treatable IEMs reported to mimic CP, belonging to 13 different biochemical categories. A further 13 treatable IEMs were included, which can present with CP-like symptoms according to expert opinion, but for which no reports in the literature were identified. For 26 of these IEMs, a treatment is available that targets the primary underlying pathophysiology (*e.g.* neurotransmitter supplements), and for the remainder (n = 41) treatment exerts stabilizing/preventative effects (*e.g*. emergency regimen). The total number of treatments is 50, and evidence varies for the various treatments from Level 1b, c (n = 2); Level 2a, b, c (n = 16); Level 4 (n = 35); to Level 4–5 (n = 6); Level 5 (n = 8). Thirty-eight (57%) of the treatable IEMs mimicking CP can be identified by ready available metabolic screening tests in blood or urine, while the remaining IEMs require more specific and sometimes invasive tests.

**Conclusions:**

Limited by the rare nature of IEMs and incomplete information in the literature, we conclude that (1) A surprisingly large number of IEMs can present with CP symptoms, as ‘CP mimics’, (2) although individually rare, a large proportion of these diseases are treatable such that neurological damage can either be reversed or prevented, (3) clinician awareness of treatable CP mimics is important for appropriate screening, diagnosis, and early intervention, and (4) systematic studies are required to elucidate the collective frequency of treatable IEMs in CP.

**Electronic supplementary material:**

The online version of this article (doi:10.1186/s13023-014-0197-2) contains supplementary material, which is available to authorized users.

## Background

Cerebral palsy (CP) is defined as a group of non-progressive disorders of movement and posture, which cause activity limitations due to disturbances that occurred in the developing fetal or infant brain [1]. CP is the most common cause of physical impairment in the pediatric population with a prevalence of 2–3 per 1000 live births [2,3]. Risk factors include prematurity, kernicterus/hyperbilirubinemia, early CNS infection, non-specific fetal or maternal infection, intra-partum asphyxia, birth trauma and intracranial hemorrhage or neonatal encephalitis [4]. However, despite advances in maternal care and obstetrical intervention in recent decades, the incidence of CP has not declined [5]. Characterization of CP is traditionally based on the predominant quality of motor impairment (spastic, dyskinetic, ataxic-hypotonic or mixed) [6], assessed on standard neurologic examination.

CP is frequently associated with cognitive, behavioral, and sensory impairments as well as epilepsy [7]. The most common morbidity, noted in approximately 40-65% of all children with CP, is intellectual developmental disability (IDD), defined by significant delay in two or more developmental domains at age less than 5 years and an intelligence quotient of ≤70 at older age. [8]. Children with co-occurring ID are at increased risk of emotional and behavioral problems [9] and other chronic health conditions requiring frequent hospitalizations [10,11] with a high burden of care and utilization of health services for individuals with CP and their families [7]. The associated medical expenditures are considerable; Kancerla *et al.* [12] showed that annual costs for children with CP exceed those of children without CP by $15,047 USD and in case of co-occurring ID by $26,617 USD. Treatment and ultimately prevention of CP (and IDD) therefore is essential to reduce the emotional and physical suffering of patients and families, and to reduce the immense health care costs.

Determination of the underlying cause of CP, whether due to a malformation, injury acquired during the pre-, peri-, or postnatal period, or a genetic aberration has obvious significance from the point of view of assessment of risk, counseling of families, and developing prevention and intervention strategies [13]. The implicit heterogeneity of CP poses a challenge for diagnosis and treatment [14], and the current management of CP follows a symptomatic approach (*e.g.*, baclofen to relieve spasticity; occupational therapy to improve mobility; pain management).

However, there are reports in the literature of inborn errors of metabolism (IEMs) that present as CP mimics, many of which are in fact amenable to therapy targeting the underlying cause that can improve neurological outcomes. IEMs are a collection of rare genetic diseases that generally result from a deficiency of an intracellular component (*e.g.*, an enzyme or transporter) of a metabolic pathway, resulting in an accumulation of a substrate or intermediate in a pathway and/or reduced ability to synthesize essential compounds. Often the central nervous system (CNS) is affected, leading to neurological disease [15]. An example is Segawa disease, also called GTPCH1-deficient dopa-responsive dystonia (GTPCH1-DRD), characterized by dystonia in childhood that is often misdiagnosed as CP *e.g.*, [16,17]. This neurotransmitter disorder can be diagnosed by standard analysis of neurotransmitter metabolites in the cerebrospinal fluid. Individuals with GTPCH1-DRD benefit from treatment with BH4 [18], amine replacement, as well as levodopa. The majority of treated individuals shows rapid clinical improvement in both CP-related symptoms (spasticity, dystonia, general tone) and are able to lead “an entirely normal life” [16].

It is currently unknown how many such treatable CP-mimics exist, as the evidence has not been systematically reviewed. We model the current review after our Treatable Intellectual Disability Endeavor (TIDE) study, which published a list of 89 treatable IEMs that present with an IDD [19], and a diagnostic algorithm with App [20]. This algorithm has been implemented in more than 400 children with unexplained IDD as part of the TIDE-BC study at the British Columbia Children’s Hospital, in Vancouver, Canada, and treatable IEMs were identified in more than 5% [20], which serves as motivation for the work presented here.

## Methods

We performed the first systematic review to compile evidence from the literature and clinical expertise of all IEMs that are known to present with CP symptoms, with a focus on those that are amenable to causal treatment. We aim to raise awareness of existence of CP mimics and formulate a diagnostic algorithm to support clinicians in the effective identification of these IEMs. We followed the Preferred Reporting Items for Systematic Reviews and Meta-Analyses (PRISMA) statement (http://www.prisma-statement.org/) [21].

### Information sources

A systematic review was performed to identify all reports of inborn errors of metabolism (IEMs) presenting with CP symptoms by searching the PubMed database, checking reference lists of relevant articles and consulting with experts in the field. We considered only articles that were published in English, described findings in humans, and those where full text publication was available electronically through our institution’s subscription. No publication date restrictions were imposed and articles included in analyses were published between 1957–2014. The last search was performed October 15, 2014.

### Definitions & Eligibility criteria

We included only studies describing diseases that are IEMs, which we have previously defined as “genetic disease involving a disorder of metabolism with confirmation based on the internationally accepted diagnostic test(s) for that IEM (gene mutations, enzyme deficiency, or specific biochemical marker); this term excludes endocrine disorders” [19]. To identify diseases most likely to be misdiagnosed as CP (*i.e.*, to exclude adult-onset), we limited our inclusion to reports where CP symptoms (Table 1) present before 5 years of age in at least one reported case.

**Table 1:** **Search strategy to systematically review treatable IEMs that present as CP mimics**

**Search strategy**			**# Treatable CP mimics**
1) PubMed search using combinations of alternative terms to describe the CP phenotype and IEMs	Definition:	Search terms:	27
	Cerebral palsy	Cerebral palsy, spastic, spasticity, dystonia, dyskinesia, ataxia, movement disorder, gait abnormalities, hypoxic(+/−ischemic) encephalopathy, perinatal asphyxia	
	Inborn error of metabolism	Inborn error of metabolism, metabolic disease, amino acids, creatine, mitochondria, neurotransmitter, organic acid, urea cycle, vitamins	
2) Manual search	Reference lists of articles identified through PubMed search were manually screened for additional citations.		15
3) Targeted search of known treatable IEMs causing ID	Diseases listed in the TIDE App (www.treatable-id.org) as treatable IEMs searched in combination with the above IEM search terms.		12
Total number of IEMs identified			54

The goal of this systematic review was to identify all treatable IEMs that present with CP symptoms, rather than to identify every report. We selected the most reliable or comprehensive reference for this publication; this implies that additional case reports of CP symptoms for a given IEM beyond those cited here may exist.

### Search strategy

The search strategy is outlined in Table 1. Primarily, combinations of search terms that describe cerebral palsy symptoms and inborn errors of metabolism were used to identify relevant articles in the PubMed database (see Table 1). Different combinations yielded common articles, indicating our approach is conservative and our search terms are effective. Results from all key word combination searches were pooled and sorted to identify 472 unique articles (*i.e.*, without duplicate results), which were then manually screened based on inclusion/exclusion criteria by the first author (ELL), with duplicate publications and those that did not meet eligibility criteria being removed. As well, reference lists of these publications were screened for additional relevant articles and these were judged for inclusion using the same criteria. Finally, to ensure that we have identified all salient IEMs, we performed another search using known treatable IEMs listed in a previous publication of treatable IEMs that present with intellectual disability (ID) [19] in combination with our search terms describing CP. Initial searches were performed independently by ELL following review protocol and all possible studies were compiled into a table. The generated list of IEMs was reviewed periodically by experts SS and CvK to confirm data, refine the search strategy and inclusion criteria.

### Data collection

The following information was extracted from each article: disease name, cerebral palsy symptoms as reported by the original authors, age of symptom onset, any treatment used (with the dose and frequency) and the treatment outcome as described by the authors. For completeness, we later assigned each disease to a biochemical category and retrieved OMIM identifiers, underlying genes and pattern of inheritance, appropriate diagnostic test, and standard of care treatment; all were agreed upon by expert clinicians. Diagnostic tests were compared with a recent diagnostic algorithm of treatable IEMs [20] and with accepted clinical practices (*e.g.*, GeneReviews®). Given the positive experience with the 2-tiered protocol for the work-up the child with IDD for treatable IEMs, we have used this as a basis for the evaluation of the child with CP for similar conditions.

### Characterizing treatment & effects

As most reviewed studies were case reports, the type of outcome measures varied. We noted whether or not treatment was implemented and the original author’s observations on treatment effect. However, due to the time span of the reviewed publications, some treatments are now available that were not at the time of the original publication. Therefore, we took a more objective approach of employing clinical expertise to determine the standard of care treatment for the systematically identified CP mimics and categorized them as treatable versus non-treatable. Causal treatment for this proportion of conditions has been thoroughly evaluated by us already [19], and we apply the same treatment recommendations here. For conditions where treatment was not previously reviewed by us, we adhered to guidelines for the specific IEMs and where needed provided expert input, with consideration for treatments applied in the original case reports of CP mimics.

Treatment of IEMs can be either ‘primary treatment’, which targets the pathophysiology at a cellular level and improves at least the CP features (muscle tone, rigidity, etc.) plus/minus the cognitive, behavioural, and MRI features; or ‘stabilizing/preventative treatment’, which halts decline and/or prevents further damage, especially during metabolic crises. For example, creatine supplementation in creatine deficiencies targets the underlying cause of the IEM and can reverse the features. Several disorders caused by deficiencies in vitamins or co-factors can show improvement in primary features with appropriate supplementation. Examples of stabilizing/preventative treatment include emergency regimen for fatty acid oxidation disorders, HSCT for lysosomal disorders and dietary restriction of amino acids in hyperhomocysteinemias and amnio acid disorders.

Non-treatable IEMs are those for which treatment of the underlying cause is not available (*i.e.*, only treatment of symptoms) or has shown to not be consistently effective. We summarize currently non-treatable IEMs in the hope that when treatment does become available, clinicians will be aware that these diseases can mimic CP and can then intervene.

### Levels of evidence

Levels of evidence for treatments were evaluated based on existing level classification by the Centre of Evidence-Based Medicine (www.cebm.net): Level 1a = Systematic Review of RCTs, 1b = Individual RCT, 1c = ’All or None’ [=(prolongation of) survival with therapy]; Level 2a = Systematic Review of Cohort Studies, 2b = Individual Cohort Study, 2c = ’Outcomes Research’ [focussed on end results of therapy for chronic conditions, including functioning and quality of life]; Level 3 = Systematic Review of Case–Control Studies; Level 4 = Individual Case–Control Study or Case-series/report; Level 5 = Expert opinion without critical appraisal; based on physiology, bench research or first principles.

### Expert-identified CP mimics

The authors who are expert clinicians in pediatric IEMs (CvK & SS) identified 13 additional diseases which they have designated as CP mimics in their clinical experience. For the majority of these, the pathophysiology is identical to IEMs in the same category for which literature evidence does exist; for example PSPH and PSAT deficiency which are both characterized by lack of serine in the brain similar to PGHDH deficiency (primarily treatable by serine supplements), or urea cycle defects in which hyperammonemic crises cause irreversible brain damage (which are preventable via dietary manipulation and medication), just like OTC deficiency for which CP-like features have been described. Likely such diseases have not (yet) been described to present as CP in the literature, likely due to the rare frequency and the increasing challenge of publishing case reports alone. The expert opinion approach was used, so that also for these IEMs, affected individuals might also receive the benefit of early detection and intervention.

### Diagnostic algorithm

To provide guidance for the identification of treatable CP-mimics, we used the 2-tiered algorithm published by van Karnebeek *et al.* [19] to identify treatable IEMs in children presenting with IDD: First tier tests are generally accessible and offered by most biochemical genetics laboratories around the world with reasonable turn-around times and affordable prices (total costs $567.97 CAD), including tests in blood (lactate; ammonia; copper; ceruloplasmin; plasma total homocysteine; plasma amino-acids, and bloodspot quantitative acylcarnitine profile) and in urine (creatine metabolites; purines and pyrimidines; organic acids; oligosaccharides; and glycosaminoglycans). Each of these screening tests has the potential to specifically identify treatable IEMs, which is then often confirmed via molecular and/or enzymatic analysis. The 2^nd^ tier requires a more directed, ‘single test per disease’ approach based on signs and symptoms. In general these tests are more invasive and more expensive.

## Results

### Treatable IEMs

Based on the defined inclusion/exclusion criteria, we identified 54 treatable IEMs reported to mimic CP (Table 1). These are categorized alphabetically into 13 biochemical categories (Table 2): amino acids (n = 5), cerebral glucose transport (n = 1), creatine (n = 1), fatty acid-related processes (n = 3), hyperhomocysteinemia (n = 2), lipids (n = 1), lysosomal (n = 4), metals (n = 2), mitochondrial (n = 3), neurotransmission (n = 9), organic acids (n = 12), urea cycle (n = 4) and vitamins/co-factors (n = 7). The CP symptoms for each IEM and corresponding reference are described in Additional file 1: Table S1. A further 13 treatable IEMs were identified by expert clinicians on our team (Table 3) for a total of 67 treatable CP mimics. Treatment modalities included: dietary restriction/supplement, co-factor/-enzyme, vitamin, substrate inhibition, substrate reduction, bone marrow and hematopoietic stem cell transplant, gene therapy. The majority of these treatments are accessible and affordable. The total number of different treatments is 50, and evidence varies for the various treatments from Level 1b, c (n = 2); Level 2a, b, c (n = 16); Level 4 (n = 35); to Level 4–5 (n = 6); Level 5 (n = 8). For 26 (39%) of these IEMs, a treatment is available that targets the primary underlying pathophysiology with the potential to improve CP symptoms; while for the remaining 41 (61%) IEMs, treatment is available that stabilizes disease or prevents further damage (*e.g.*, treatment of Succinic semi-aldehyde dehydrogenase deficiency (SSADH) with Vigabatrin can stabilize symptoms [22,23]).

**Table 2:** **Overview of all 54 treatable IEMs presenting as CP mimics identified through systematic literature review**

**Biochemical category**	**Disease name**	**OMIM#**	**Gene(s)**	**Treatment**	**Effect**	**Level of evidence**	**CP symptoms**
Amino acids	Hartnup disease	234500	*SLC6A19 (AR)*	High protein diet	Stabilizing/preventative treatment	4-5	Dystonia
	Hyperornithinemia-hyperammonemia-homocitrullinuria (HHH) syndrome	238970	*SLC25A15 (AR)*	Dietary protein restriction, ornithine supplement, sodium benzoate, phenylacetate	Stabilizing/preventative treatment	4	Spasticity
	Late onset non-ketotic hyperglycinemia	605899	*AMT/GLDC/GCSH (AR)*	Glycine restriction, +/− sodium benzoate, NMDA receptor antagonists, other neuromodulating agents	Stabilizing/preventative treatment	4-5	Spastic diplegia
	Phenylketonuria (PKU)	261600	*PAH (AR)*	Dietary phenylalanine restriction +/− amino acid supplements (BH(4) supplement)	Stabilizing/preventative treatment	2a (4)	Spastic diplegia
	PHGDH deficiency (Serine deficiency)	601815	*PHGDH (AR)*	L-serine & +/−glycine supplements	Primary/targeting underlying pathophysiology	4	Spastic diplegia/tetraparesis
Cerebral glucose transport	Blood brain-barrier glucose-transporter (GLUT1) defect	606777	*SLC2A1 (AR)*	Ketogenic diet	Primary/targeting underlying pathophysiology	4	Spasticity, dystonia, ataxia
Creatine	GAMT deficiency	612736	*GAMT (AR)*	Arginine restriction, creatine & ornithine supplements	Primary/targeting underlying pathophysiology	4	Movement disorder: extrapyramidal signs, athetosis, & ataxia
Fatty acid oxidation	Medium-chain acyl-coenzyme A dehydrogenase (MCAD) deficiency	201450	*ACADM (AR)*	Emergency regimen, L-carnitine, avoid fasting	Stabilizing/preventative treatment	2a	CP symptoms
	Short-chain acyl-CoA dehydrogenase (SCAD) deficiency	201470	*ACADS (AR)*	Emergency regimen, L-carnitine	Stabilizing/preventative treatment	2c	Spastic diplegia
	Very-long-chain acyl-CoA dehydrogenase (VLCAD) deficiency	201475	*ACADVL (AR)*	Avoidance of fasting, low-fat diet, Medium Chain Triglyceride oild	Stabilizing/preventative treatment	4	Neonatal asphyxia
Hyperhomo-cysteinemia	Homocystinuria due to Cystathionine β-synthase deficiency	236200	*CBS (AR)*	Methionine restriction, +/− pyridoxine, +/− betaine	Stabilizing/preventative treatment	2c	Dystonia
	MTHFR deficiency	236250	*MTHFR (AR)*	Betaine supplements, +/− folate, carnitine, methionine supplements	Stabilizing/preventative treatment	4	Ataxic gait, hypotonia, extrapyramidal movements, upper motor neuron signs
Lipids	Abetalipoproteinemia	200100	*MTTP (AR)*	Low long-chain fatty acid diet with fat-soluble vitamin (*i.e*., A, D, E, K) supplementation	Primary/targeting underlying pathophysiology	4	Ataxia, abnormal gait
Lysosomal	Fucosidosis	230000	*FUCA1 (AR)*	Haematopoietic stem cell transplant	Stabilizing/preventative treatment	5	Severe spasticity; spastic paresis, generalized dystonia
	Krabbe disease	245200	*GALC (AR)*	Haematopoietic stem cell transplant	Stabilizing/preventative treatment	2c	Progressive spasticity
	Metachromatic leucodystrophy (MLD)	250100	*ARSA (AR)*	Haematopoietic stem cell transplant	Stabilizing/preventative treatment	4-5	Loss of all gross motor function measured by CP scale; ataxia
	Neimann-Pick, type C	257220	*NPC1, NPC2*	Miglustat	Stabilizing/preventative treatment	1b	Axial hypotonia, spastic diparesis, dystonic posturing of the hands
Metals	Menkes Disease	309400	*ATP7A*	Copper histidine	Stabilizing/preventative treatment	4	Progressive spasticity, hypotonia
	Wilson Disease	277900	*ATP7B*	Zinc & tetrathiomolybdate; oxcarbazepine	Stabilizing/preventative treatment	1b	Neurological symptoms, dystonia
Mitochondria	Coenzyme Q10 deficiency	607426	*COQ2, APTX, PDSS1, PDSS2, CABC1, COQ9 (most AR)*	CoQ supplements	Primary/targeting underlying pathophysiology	4	Spastic paresis; progressive ataxia and dystonia
	MELAS	540000	*mt.A3243G, mt.G13513A (mtDNA)*	Arginine supplements	Stabilizing/preventative treatment	4-5	dx. CP
	Pyruvate dehydrogenase deficiency	312170, 245348	*PDHA1 (X-linked recessive), DLAT (AR), PDHX (AR)*	Ketogenic diet & thiamine	Primary/targeting underlying pathophysiology	4	Spastic quadriplegia; dystonia
Neurotransmission	Aromatic-L-amino-acid decarboxylase deficiency	608643	*DDC (AR)*	MAO inhibitors, B6, anti-cholinergics, dopa agonists)	Primary/targeting underlying pathophysiology	4	Limb dystonia, athetoid movement
	DHPR deficiency (biopterin deficiency)	261630	*QDPR (AR)*	BH4, diet, amine replacement, folinic acid	Primary/targeting underlying pathophysiology	4	Ataxia, gait disorder, peripheral spasticity
	Dopamine transporter deficiency syndrome	126455	*SLC6A3*	Dopamine antagonist (Ropinirole)	Primary/targeting underlying pathophysiology	4	dx. CP
	GTPCH1-deficient dopa-responsive dystonia (aka Segawa’s disease)	233910	*GCH1 (AR)*	BH4, amine replacement	Primary/targeting underlying pathophysiology	4	dx. CP; spastic diplegia
	PTPS deficiency (biopterin deficiency)	261640	*PTS (AR)*	BH4, diet, amine replacement	Primary/targeting underlying pathophysiology	4	Dystonia; spastic extremities; generalized dystonia, choreoathetoid arm movements & axial hypotonia
	Sepiapterin reductase deficiency	612716	*SPR (AR)*	Amine replacement	Primary/targeting underlying pathophysiology	4	Limb spasticity, dystonic signs; “hypotonic cerebral palsy”; dystonia, axial hypotonia; misdx. CP
	Succinic semialdehyde dehydrogenase deficiency (SSADH)	271980	*ALDH5A1 (AR)*	Vigabatrin	Stabilizing/preventative treatment	4	Hypotonia, ataxia; gait clumsiness, dystonia
	Tyrosine hydroxylase deficiency	605407	*TH (AR)*	L-dopa substitution	Primary/targeting underlying pathophysiology	4	Spastic paraplegia/tetraparesis
	Vesicular monoamine transporter 2 (VMAT2)	193001	*SLC18A2*	Dopamine aginist	Primary/targeting underlying pathophysiology	4	Dystonia
Organic acids	β-Ketothiolase deficiency	203750	*ACAT1 (AR)*	Avoid fasting, emergency regimen, protein restriction	Stabilizing/preventative treatment	5	Ataxia, diplegia, hypotonia
	2-Methyl-3-hydroxybutyryl-CoA dehydrogenase (MHBD) deficiency	300438	*HSD17B10 (X-linked)*	Avoid fasting, emergency regimen, isoleucine restricted diet	Stabilizing/preventative treatment	5	Ataxia, dystonia, choreoathetosis, spastic di-/tetra-plegia, hypotonia
	3-Methylcrotonyl-CoA carboxylase (MCC) deficiency	210200; 210210	*MCC1/MCC2 (AR)*	Dietary protein restriction; carnitine, glycine, biotin supplements; avoid fasting; emergency regimen	Stabilizing/preventative treatment	5	dx. CP
	3-Methylglutaconic aciduria type 1	250950	*AUH (AR)*	Carnitine supplements, avoid fasting, emergency regimen	Stabilizing/preventative treatment	5	dx. CP
	Ethylmalonic encephalopathy	602473	*ETHE1 (AR)*	N-acetylcysteine, oral metronidazol	Stabilizing/preventative treatment	4	CNS malformations, episodic ataxia; pyramidal tract signs
	Glutaric aciduria type I (GA1) aka glutaryl-CoA dehydrogenase deficiency	231670	*GCDH (AR)*	Lysine restriction, carnitine supplements	Stabilizing/preventative treatment	2a	Generalized spasticity, dystonia with athethosis; dx. CP; dyskinesia, dystonic tetraparesis
	Isovaleric acidemia	243500	*IVD (AR)*	Dietary protein restriction, carnitine supplements, avoid fasting, emergency regimen	Stabilizing/preventative treatment	2c	Hypotonia, paresis
	Multiple acyl-CoA-dehydrogenase deficiency (MADD) (aka Glutaric aciduria type II)	231680	*ETFA, ETFB, ETFDH (AR)*	Carnitine, riboflavin, β-hydroxybutyrate supplements; emergency regimen	Primary/targeting underlying pathophysiology	5	Encephalopathy
	Maple syrup urine disease	248600	*DBT, BCKDHB, BCKDHA (AR)*	Dietary restriction, branched amino-acids, avoid fasting, (liver transplantation)	Stabilizing/preventative treatment (liver tx = primary treatment)	4 (4)	Spastic diplegic CP; paroxysmal dystonia; ataxia
	Methylmalonic acidemia (mutase deficiency)	251000	*MUT (AR)*	Dietary protein restriction, carnitine supplements, avoid fasting, emergency regimen	Stabilizing/preventative treatment	2c	Total body dystonia
	Lesch-Nyhan syndrome	300322	*HPRT1 (X-linked)*	Haematopoietic stem cell transplant	Primary/targeting underlying pathophysiology	4-5	dx. Athetotic/dyskinetic CP; dystonia
	Propionic acidemia	606054	*PCCA, PCCB (AR)*	Dietary protein restriction, carnitine supplements, avoid fasting, emergency regimen	Stabilizing/preventative treatment	2c	Dystonia, hypotonia
Urea cycle	Argininemia	207800	*ARG1 (AR)*	Dietary protein restriction, arginine supplement, sodium benzoate, phenylbutyrate (Liver transplantation)	Stabilizing/preventative treatment (liver tx = primary treatment)	2b (4)	Spastic diplegia, ataxia, dx. CP
	Argininosuccinic aciduria	207900	*ASL (AR)*	Low protein diet, arginine-supplements, sodium benzoate, phenylbutyrate (liver transplantation)	Stabilizing/preventative treatment (liver tx = primary treatment)	2b (4)	Cerebellar ataxia
	Citrullinemia, type II	605814	*SLC25A13 (AR)*	Dietary protein restriction, arginine supplement, sodium benzoate, phenylbutyrate (Liver transplantation)	Stabilizing/preventative treatment (liver tx = primary treatment)	2b (4)	dx. CP; spastic quadriplegia
	Ornithine transcarbamylase deficiency	311250	*OTC (X-linked)*	Dietary protein restriction, citrulline supplements, sodium benzoate/phenylbutyrate (Liver transplantation)	Stabilizing/preventative treatment (liver tx = primary treatment)	2b (4)	Hemiplegia; ataxia; gait disturbance
Vitamins/Co-factors	Biotinidase deficiency	2532760	*BTD (AR)*	Biotin supplement	Primary/targeting underlying pathophysiology	2c	Spastic tetraparesis
	Biotin-thiamine-responsive basal ganglia disease	607483	*SLC19A3 (AR)*	Biotin supplement	Primary/targeting underlying pathophysiology	4	Ataxia, dystonia
	Cerebral folate deficiency syndrome	613068	*FOLR1 (AR)*	Folinic acid	Primary/targeting underlying pathophysiology	4	spastic paraplegia; perinatal asphyxia
	Holocarboxylase synthetase deficiency	253270	*HLCS (AR)*	Biotin supplement	Primary/targeting underlying pathophysiology	4	dx. CP
	Hypermanganesemia with dystonia, polycythemia, and cirrhosis (HMDPC)	613280	*SLC30A10 (AR)*	Chelation therapy	Primary/targeting underlying pathophysiology	4	Dystonia
	Molybdenum cofactor deficiency	252150	*MOCS1, MOCS2, (AR)*	Precursor Z/cPMP	Primary/targeting underlying pathophysiology	4	Spastic quadriplegia dx. CP
	Pyridoxamine 5’-phosphate oxidase deficiency	610090	*PNPO (AR)*	Pyridoxal 5’-phosphate	Stabilizing/preventative treatment	4	Spastic quadriplegia

*Emergency regimen* is defined as: Adjustment in management of a particular IEM to prevent or minimize metabolic decompensations (and related complications) during illness, periods of decreased intake or increased energy demand. The mainstay includes high caloric intake, generous fluid management (oral, tube or intravenous), addition/increase of vitamins/co-factors or medications, along with avoidance of substances which cannot be metabolized in patients with this IEM [24].

The IEMs are grouped according to the biochemical phenotype as presented in standard textbooks, and alphabetically.

**Table 3:** **Overview of all 13 treatable IEMs presenting as CP mimics identified by clinical experts on our team**

**Biochemical category**	**Disease name**	**OMIM#**	**Gene(s)**	**Treatment**	**Effect**	**Level of evidence**
Amino acids	PSAT deficiency	610992	PSAT1 (AR)	L-serine & +/−glycine supplements	Primary/targeting underlying pathophysiology	4
	PSPH deficiency (Serine deficiency)	614023	PSPH (AR)	L-serine & +/−glycine supplements	Primary/targeting underlying pathophysiology	4
Creatine	Arginine:glycine amidinotransferase (AGAT) deficiency	612718	*GATM (AR)*	Creatine supplements	Primary/targeting underlying pathophysiology	4
	Creatine transporter deficiency	300352	*SLC6A8 (X-linked)*	Creatine, glycine, arginine supplements	Primary/targeting underlying pathophysiology	4
Fatty acid oxidation	Carnitine palmitoyltransferase I deficiency	255120	CPT1A (AR)	Low-fat, high carbohydrate diet, avoid fasting, Medium Chain Triglyceride oil	Stablizilng/preventative treatment	4
Hyperhomocystinuria	Cobalamin deficiencies (*e.g.*, C, D, E, F, G)	251110, 277400, 277410, 236270, 277380	MMACHC, MMADHC, MTRR, LMBRD1, MTR (AR)	Hydroxy-/cyanocobalamin (+/− diet restriction, betaine, B12)	Stabilizing/preventative treatment	4
Lipid storage (Leukodystrophy)	Cerebrotendinous xanthomatosis (CTX)	213700	CYP27A1 (AR)	Chenodeoxycholic acid	Stabilizing/preventative treatment	4
Organic acids	HMG-CoA lyase deficiency	246450	HMGCL (AR)	Protein restriction, avoid fasting, emergency regimen	Stabilizing/preventative treatment	4-5
	mHMG-CoA synthase deficiency	605911	HMGCS2 (AR)	Avoid fasting,emergency regimen, +/−dietary precursor restriction	Stabilizing/preventative treatment	5
	SCOT deficiency	245050	OXCT1 (AR)	Avoid fasting, protein restriction, emergency regimen	Stabilizing/preventative treatment	5
Urea cycle	Carbamoyl phosphate synthetase (CPS) deficiency	237300	*CPS1 (AR)*	Dietary protein restriction, arginine supplement, sodium benzoate, phenylbutyrate (Liver transplantation)	Stabilizing/preventative treatment (primary/targeting underlying pathophysiology)	2b (4)
	Citrullinemia type I (ASS deficiency)	215700	*ASS1 (AR)*	Dietary protein restriction, arginine supplement, sodium benzoate, phenylbutyrate (Liver transplantation)	Stabilizing/preventative treatment (primary/targeting underlying pathophysiology)	2b (4)
	N-acetyl-glutamate synthetase deficiency	237310	*NAGS (AR)*	Dietary protein restriction, arginine supplement, sodium benzoate, phenylbutyrate (Liver transplantation)	Stabilizing/preventative treatment	4

The IEMs are grouped according to the biochemical phenotype as presented in standard textbooks, and alphabetically.

Thirty-eight of the 67 disorders (57%) of the treatable IEMs described in this review can be identified by ‘1^st^ tier’ metabolic screening tests in blood or urine (Table 4). The other 29 (43%) require more specific and sometimes invasive methods (‘2^nd^ tier tests’; Table 5). Of the 1^st^ tier tests in the Treatable IDD protocol, urine MPS and urine oligosaccharides are not required for the diagnostic evaluation of CP for treatable IEMs. Most 1^st^ tier tests which detect treatable IEMs described in the literature as CP mimics can also identify ‘treatable IEMs identified by expert opinion’ (*e.g.*, urine creatine metabolites identify GAMT deficiency, but also Creatine transporter deficiency and AGAT deficiency).

**Table 4:** **Summary of all treatable IEMs (n = 38, 57%) that can be detected by ‘1** ^**st**^ **-tier’ metabolic screening tests, which are affordable and accessible, with the potential to identify multiple IEMs**

**Blood tests**			
Acylcarnitine profiles (n = 3)	● MCAD deficiency		
	● SCAD deficiency		
	● VLCAD deficiency		
Free-to-total serum/plasma carnitine (n = 1)	● Carnitine palmitoyltransferase I deficiency		
Plasma Amino Acids (n = 10)	● Argininemia	● Hartnup disease	● MTHFR Deficiency (&tHcy)
	● Argininosuccinate lyase deficiency	● HHH syndrome	● Ornithine transcarbamylase deficiency
	● Citrullinemia type I	● Maple syrup urine disease	● Phenylketonuria (PKU)
	● Citrullinemia, type II		
Plasma cholesterol (n = 1)	● Cerebrotendinous xanthomatosis (CTX)		
Serum copper & ceruloplasmin (n = 2)	● Menkes Disease (& urine deoxypyridinoline)		
	● Wilson Disease (& urine copper)		
**Urine tests**			
Urine creatine metabolites (n = 1)	● GAMT deficiency		
Urine oligosaccharides (n = 1)	● Fucosidosis		
Urine organic acids (n = 17)	● 3-Methylglutaconic aciduria type 1	● Ethylmalonic encephalopathy (& ACP)	● MHBD deficiency
	● 3-Methylcrotonyl-CoA carboxylase (MCC) deficiency (& ACP)	● SSADH	● HMG-CoA lyase deficiency
	● β-Ketothiolase deficiency	● Glutaric aciduria type I	● mHMG-CoA synthase deficiency
	● Cobalamin deficiencies (& PAA)	● Holocarboxylase synthetase deficiency	● Multiple acyl-CoA-dehydrogenase deficiency (MADD)
	● Cystathionine β-synthase deficiency	● Isovaleric academia	● Propionic academia
		● Methylmalonic academia	● SCOT deficiency
Urine purines & pyrimidines (n = 2)	● Lesch-Nyhan syndrome		
	● Molybdenum cofactor deficiency		

*Abbreviations* include: *ACP* acylcarnitine profiles, *tHcy* total homocystine, *PAA* plasma amnio acids.

**Table 5:** **All IEMs (n = 29, 43%) requiring a specific ‘2** ^**nd**^ **-tier’ test for diagnosis**

**Biochemical category**	**Disease**	**Diagnostic test**
Amino acids	PSAT deficiency	CSF amino acids (& PAA)
	PSPH deficiency (Serine deficiency)	CSF amino acids (& PAA)
	Late onset non-ketotic hyperglycinemia	CSF AA (& Plasma AA)
	PHGDH deficiency (Serine deficiency)	CSF AA (& Plasma AA)
Cerebral glucose transport	Blood brain-barrier glucose-transporter (GLUT1) defect	CSF glucose:plasma glucose ratio
Creatine	Arginine: glycine amidinotransferase (AGAT) deficiency	*GATM* gene sequencing
	Creatine transporter deficiency	*SLC6A8* gene sequencing
Lipids	Abetalipoproteinemia	CBC smear, stool samples, fasting lipid profile, *MTTP* gene analysis
Lysosomal	Krabbe disease	WBC enzyme testing
	Metachromatic leucodystrophy (MLD)	Arylsulfatase-A enzyme activity
	Niemann-Pick, type C	Filipin staining test (fibroblasts) & *NPC1/NPC2* gene analyses
Mitochondria	Coenzyme Q10 deficiency	Coenzyme Q10 (fibroblasts) & gene(s) analysis
	MELAS	Mitochondrial DNA mutation testing
	Pyruvate dehydrogenase deficiency	Blood & CSF lactate:pyruvate ratio (enzyme activity, gene(s) analysis)
Neurotransmission	Aromatic-L-amino-acid decarboxylase deficiency	CSF biogenic amines
	DHPR deficiency (biopterin deficiency)	CSF neurotransmitters & biopterin loading test
	Dopamine transporter deficiency syndrome	CSF neurotransmitters
	GTPCH1-deficient dopa-responsive dystonia	CSF neurotransmitters & biopterin/Phe loading test; clinical trial of L-dopamine, GTCPH gene analysis
	PTPS deficiency (biopterin deficiency)	CSF neurotransmitters & biopterin loading test
	Sepiapterin reductase deficiency	CSF neurotransmitters & biopterin/Phe loading test
	Tyrosine hydroxylase deficiency	CSF neurotransmitters & *TH* gene analysis
	Vesicular monoamine transporter 2 (VMAT2)	CSF monoamine metabolites
Urea cycle	Carbamoyl phosphate synthetase (CPS) deficiency	*CPS* gene analysis
	N-acetyl-glutamate synthetase deficiency	*NAGS* gene analysis
Vitamins/Co-factors	Biotinidase deficiency	Biotinidase enzyme activity
	Biotin-thiamine-responsive basal ganglia disease	*SLC19A3* gene analysis
	Cerebral folate deficiency syndrome	CSF tetrahydrofolate
	Hypermanganesemia with dystonia, polycythemia, and cirrhosis	Whole-blood manganese concentrations, *SLC30A10* gene analysis
	Pyridoxamine 5’-phosphate oxidase deficiency	Plasma, CSF

The IEMs are listed per biochemical category, with the specific biochemical/genetic diagnostic test per disease. *Abbreviations* include: *CSF* cerebrospinal fluid, *PAA* plasma amnio acids, *Phe* phenylalanine.

### Non-treatable IEMs

There are several IEMs that can present as CP mimics that are not (yet) treatable. These include:

Disorders of amino acids (*e.g.*, Hyperprolinemia type I); cholesterol (*e.g.*, Mevalonic aciduria); lipids (*e.g.*, FAHN, Pelizaeus-Merzbacher disease); lysosomal disorders, such as sphingolipidoses (*e.g.*, GM1/2 gangliosidoses); mitochondrial diseases (*e.g.*, Leigh’s disease, sulfite oxidase deficiency, respiratory chain deficiencies); metals (*e.g.*, NBIA1, PLAN); organic acids (*e.g.*, Fumarate Hydratase Deficiency); peroxisomes (*e.g.*, NALD); and purine and pyrimidine disorders (*e.g.*, Adenylosuccinase deficiency, Purine nucleoside phosphorylase deficiency). Some of these conditions have emerging treatments, but not an established standard of care treatment; for example, Canavan disease, [25] and Gaucher disease, type 3 [26].

## Discussion

To our knowledge, this is the first comprehensive literature review to extensively review and compiled all the known cases of treatable IEMs with co-occurring CP-like symptoms (dystonia, movement disorder, basal ganglia lesions, etc. before age 5 years). A surprisingly high number of CP mimics were identified, totaling 67 treatable IEMs (54 evidence-based, 13 expert-identified) and 43 non-treatable IEMs.

Among the treatable IEMs, we made the distinction between treatments that address primary causes of CP symptoms versus more secondary causes. For conditions that are primarily treatable, treatment targets the underlying pathophysiology and is most effective. For example, the neurotransmitter defect Tyrosine hydroxylase (TH) deficiency is highly amenable to early intervention treatment with L-dopa shows dramatic improvement and reversal of symptoms [27,28]. In diseases with secondary causes of CP symptoms (*e.g.*, MCADD, MSUD, organic academia, urea cycle deficiency), metabolic crises such as hypoglycemia or acidosis caused by the metabolic defect can lead to neurologic sequelae mimicking CP. For these disorders prevention or stabilization, via emergency regimen, medical diets, etc., is best possible outcome.

Several IEMs presenting as CP mimics can be identified with minimally invasive testing. For example in biotinidase deficiency, the lack of the biotinidase enzyme causes accumulation of organic acid metabolites leading to ketolactic acidosis and hyperammonemia which can develop CP-like neurological manifestation (*e.g.*, seisures, hypotonia, ataxia, feeding problems, cognitive developmental delay, etc.) [29]. Diagnosis requires minimally invasive testing (blood sampling for serum enzyme activity) and many of these symptoms can be alleviated following biotin supplementation and permanent neurological deficits such as optic atrophy, hearing loss and/or IDD may be prevented if treated early [29].

Non-treatable IEMs were also reported with the hope that new treatments might become available in the future. For example, experimental treatments are currently being explored in trials for Pantothenate kinase 2-associated neurodegeneration (PKAN, also known as Hallervorden-Spatz disease), such as gene therapy, chelation with Deferiprone [30] to prevent neurodegeneration caused by brain iron accumulation.

Although 48% of the IEMs listed can be identified by newborn screening (NBS) in most Canadian provinces [31], NBS is not universally standardized; also some diseases or very mild cases are missed. Therefore, these treatable IEMs should not be excluded from a differential diagnosis and are important to look for as part of clinical investigations for CP. There are also be other non-IEM disorders that can present with CP symptoms (*e.g*. endocrine disorders [32]), which may be useful for the clinician to be aware.

Whole exome and genome sequencing allows for detection of new CP mimics and, along with other metabolomics approaches and enhanced neuroimaging, will facilitate research into the phenotypic spectrum and underlying pathophysiology of these disorders. In the future, screening for such conditions might be done by whole exome sequencing, with targeted analysis of the atypical CP genes, followed by biochemical confirmation for the IEMs listed here. However, it must be emphasized that clinical history and exam remain key in the interpretation of genomic data [33]. Furthermore the lumbar puncture, although invasive, should not be avoided as it allows for CSF neurotransmitter analysis, which is highly sensitive and often guides the clinician in further diagnostic and therapeutic decisions.

Despite our attempts to be as thorough as possible in this systematic review, we acknowledge the limitations of our study. Many of the IEMs listed are very rare diseases, with incidence ranging 1:10,000 (PKU) to 1:250,000 or less (GAMT deficiency), and thus, the number of publications is relatively low and evidence for treatments is sometimes sparse. As well, it can be difficult to publish case reports, which could contribute to a lack of literature evidence and preclude inclusion from our study. We have attempted to account for this by including expert clinician experience to identify IEMs that are not yet described in the literature. Despite our efforts to be as inclusive as possible when compiling the ‘expert’ list based on a working knowledge of IEMs that mimic CP (Table 3), we acknowledge that some of the potential candidates may have been omitted. Finally, neurologic symptoms are often insufficiently described in metabolic case reports, with focus often on the biochemical features of an IEM; this combined with the broad usage of the term of CP (and its different forms), the classification of an IEM phenotype as ‘CP mimic’ was challenging and depended on the authors’ expertise.

The extensive number of distinct IEMs that may mimic CP, each requiring particular diagnostic tests, places a significant information burden on clinicians. Here we have gone to extensive lengths to compile all known IEMs mimicking CP with the hope to help raise awareness and facilitate diagnostic approach with an established algorithm [19]. This is by no means meant as directive but rather, as a supportive tool to the clinician managing children with CP-like symptomatology. Symptoms which should prompt the clinician to search for an underlying IEM or other neurogenetic defect include -but not limited to- the following ‘red flags’: normal MRI findings imaging; abnormalities isolated to the globus pallidus; severe symptoms in the absence of a history of perinatal injury; a pattern of disease inheritance, or consanguinity; neurodevelopmental regression, or progressively worsening symptomatology; isolated muscular hypotonia; rigidity (as opposed to spasticity) on physician examination; paraplegia [33].

Early detection of treatable IEMs and timely intervention is of the utmost importance in order to prevent future brain insult and manifestation of CP symptoms. Additionally, the determination of the underlying cause of CP, whether treatable or not, has significance from the point of view of risk assessment, counselling for families, improved access to community services, better management of co-morbidities, and the development of prevention and intervention strategies [13]. This would not only spare suffering of individuals, but would have broader impact in terms of alleviating the economic and social burden of CP as well.

As with the TIDE approach of systematic screening [19], it is our hope that the use of this algorithm will provide more insight into frequency of IEMs amongst the CP population, and further increase our understanding of the etiology of CP. Most importantly, early diagnosis of IEMs will allow initiation of causal treatment to improve outcomes via the reduction of possibly prevention of the the physical burdens of CP.

## Conclusions

We provided the first systematic review of treatable IEMs that can present with symptoms of CP. There are many single such reports in the literature, however, the collective incidence of treatable IEM mimicking CP is unknown and can be determined only by systematic or large-scale screening studies. Increasing clinician awareness might be worthwhile, as with timely diagnosis and appropriate treatment, these conditions can show improvement in the primary features, or stabilization and prevention of further neurologic sequelae and decline. The usefulness of our diagnostic algorithm remains to be determined but represents a first step towards increased recognition of potentially treatable conditions in the child with CP.

## Additional file

Additional file 1: Table S1. Overview of all 50 treatable IEMs presenting as CP mimics identified through systematic literature review. The IEMs are grouped according to the biochemical phenotype as presented in standard textbooks, and alphabetically.
