# Supplementary material for: Treatable inborn errors of metabolism presenting as cerebral palsy mimics: systematic literature review
Source: Orphanet J Rare Dis. 2014 Nov 30;9:197. doi: 10.1186/s13023-014-0197-2 (PMC4273454; doi:10.1186/s13023-014-0197-2)
Supplement: Additional file 1: Table S1. — Overview of all 50 treatable IEMs presenting as CP mimics identified through systematic literature review. The IEMs are grouped according to the biochemical phenotype as presented in standard textbooks, and alphabetically. [file 13023_2014_197_MOESM1_ESM.pdf]

Treatable inborn errors of metabolism presenting as cerebral palsy mimics: Systematic literature review

Emma L. Leach, MSc (emma.leach@cw.bc.ca)[1,2], Michael Shevell, MD, CM, FRCPC (michael.shevell@muhc.mcgill.ca)[3,4], Kristin Bowden, PhD (kristinbowden10@gmail.com)[2], Sylvia Stockler-Ipsirogly, MD, PhD (sstockler@cw.bc.ca)[2,5,6], Clara D.M. van Karnebeek, MD, PhD, FCCMG (cvankarnebeek@cw.bc.ca) [2,5,6,7*]

Affiliations:

1. Provincial Medical Genetics Program, B.C. Women's Hospital;

2. University of British Columbia, Vancouver, B.C., Canada;

3. Department of Pediatrics, Montreal Children's Hospital, McGill University, Montreal, Canada;

4. Department of Neurology/Neurosurgery, Montreal Children's Hospital, McGill University, Montreal, Canada;

5. Division of Biochemical Diseases, Department of Pediatrics, B.C. Children's Hospital, Vancouver, Canada;

6. Treatable Intellectual Disability Endeavour in British Columbia (www.tidebc.org), Vancouver, Canada;

7. Centre for Molecular Medicine and Therapeutics; Child and Family Research Institute, Vancouver, Canada;

*Corresponding author (Dr. C. van Karnebeek): Biochemical Diseases, Department of Pediatrics, UBC, Rm K3-201, 4480 Oak Street, Vancouver, BC V6H 3V4, Canada. Tel: +1 604-875-2628 / Fax: +1 604 875 2349. Email address: cvankarnebeek@cw.bc.ca.

Supplementary Data

Table S1. Overview of all 50 treatable IEMs presenting as CP mimics identified through systematic literature review. The IEMs are grouped according to the biochemical phenotype as presented in standard textbooks, and alphabetically.

Biochemical category	Disease name	OMIM#	Gene(s)	Treatment	Effect	Level of Evidence	CP symptoms	CP mimic reference
Amnio acids	Hartnup disease	234500	*SLC6A19 (AR)*	High protein diet	stabilizing/ preventative treatment	IV	Dystonia	[30]
	Hyperornithinemia-hyperammonemia-homocitrullinuria (HHH) syndrome	238970	*SLC25A15 (AR)*	Dietary protein restriction, ornithine supplement, sodium benzoate, phenylacetate	stabilizing/ preventative treatment	IV	Spasticity	[31, 32]
	Late onset non-ketotic hyperglycinemia	605899	*AMT/GLDC/ GCSH (AR)*	Glycine restriction, +/- sodium benzoate, NMDA receptor antagonists, other neuromodulating agents	stabilizing/ preventative treatment	IV	Spastic diplegia	[33]
	Phenylketonuria (PKU)	261600	*PAH (AR)*	Dietary phenylalanine restriction +/- amino acid supplements (BH(4) supplement)	stabilizing/ preventative treatment	II	Spastic diplegia	[34, 35]
	PHGDH deficiency (Serine deficiency)	601815	*PHGDH (AR)*	L-serine & +/[illegible]glycine supplements	primary / targeting underlying pathophysiology	IV	Spastic diplegia/ tetraparesis	[36, 37]
Cerebral glucose transport	Blood brain-barrier glucose-transporter (GLUT1) defect	606777	*SLC2A1 (AR)*	Ketogenic diet	primary / targeting underlying pathophysiology	IV	Spasticity, dystonia, ataxia	[38]
Creatine	GAMT deficiency	612736	*GAMT (AR)*	Arginine restriction, creatine & ornithine supplements	primary / targeting underlying pathophysiology	IV	Movement disorder: extrapyramidal signs, athetosis, & ataxia	[39, 40]
Fatty acid oxidation	Medium-chain acyl-coenzyme A dehydrogenase (MCAD) deficiency	201450	*ACADM (AR)*	Sick day formula, L-carnitine, avoid fasting	stabilizing/preventative treatment	III	CP symptoms	[41]
	Short-chain acyl-CoA dehydrogenase (SCAD) deficiency	201470	*ACADS (AR)*	Sick day formula, L-carnitine	stabilizing/preventative treatment	III	Spastic diplegia	[42]

**Biochemical category**	**Disease name**	**OMIM#**	**Gene(s)**	**Treatment**	**Effect**	**Level of Evidence**	**CP symptoms**	**CP mimic reference**
Hyperhomo-cysteinemia	Homocystinuria due to Cystathionine [illegible]-synthase deficiency	236200	*CBS (AR)*	Methionine restriction, +/- pyridoxine, +/- betaine	stabilizing/preventative treatment	III	Dystonia	[43, 44]
	MTHFR Deficiency	236250	MTHFR (AR)	Betaine supplements, +/- folate, carnitine, methionine supplements	stabilizing/preventative treatment	IV	Ataxic gait, hypotonia, extrapyramidal movements, upper motor neuron signs	[45]
Lysosomal	Fucosidosis	230000	*FUCA1 (AR)*	Haematopoietic stem cell transplant	stabilizing/preventative treatment	IV	Severe spasticity; spastic paresis, generalized dystonia	[46, 47]
	Krabbe disease	245200	*GALC (AR)*	Haematopoietic stem cell transplant	stabilizing/preventative treatment	III	Progressive spasticity	[48]
	Metachromatic leucodystrophy (MLD)	250100	*ARSA (AR)*	Haematopoietic stem cell transplant	stabilizing/preventative treatment	IV	Loss of all gross motor function measured by CP scale	[49]
	Neimann-Pick, type C	257220	*NPC1, NPC2*	Miglustat	stabilizing/preventative treatment	I	Axial hypotonia, spastic diparesis, dystonic posturing of the hands	[50]
Metals	Menkes Disease	309400	*ATP7A*	Copper histidine	stabilizing/preventative treatment	IV	Progressive spasticity, hypotonia	[51]
	Wilson Disease	277900	*ATP7B*	Zinc & tetrathiomolybdate; oxcarbazepine	stabilizing/preventative treatment	III	Neurological symptoms, dystonia	[52]

Biochemical category	Disease name	OMIM#	Gene(s)	Treatment	Effect	Level of Evidence	CP symptoms	CP mimic reference
Mitochondria	Coenzyme Q10 deficiency	607426	*COQ2, APTX, PDSS1, PDSS2, CABC1, COQ9 (most AR)*	CoQ supplements	primary / targeting underlying pathophysiology	IV	Spastic paresis; progressive ataxia and dystonia	[53, 54]
	MELAS	540000	*mtDNA 8993 (NARP)*	Arginine supplements	stabilizing/preventative treatment	IV	dx. CP	[55]
	Pyruvate dehydrogenase deficiency	312170, 245348	*PDHA1 (X-linked recessive), DLAT (AR), PDHX (AR)*	Ketogenic diet & thiamine	primary / targeting underlying pathophysiology	IV	Spastic quadriplegia; dystonia	[56, 57, 58]
Neurotransmission	Aromatic-L-amino-acid decarboxylase deficiency	608643	*DDC (AR)*	MAO inhibitors, B6, anti-cholinergics, dopa agonists)	primary / targeting underlying pathophysiology	IV	Limb dystonia, athetoid movement	[59, 60]
	DHPR deficiency (biopterin deficiency)	261630	*QDPR (AR)*	BH4,diet, amine replacement, folinic acid	primary / targeting underlying pathophysiology	IV	Ataxia, gait disorder, peripheral spasticity	[61]
	Dopamine transporter deficiency syndrome	126455	*SLC6A3*	Dopamine antagonist (Ropinirole)	primary / targeting underlying pathophysiology	IV	dx. CP	[62]
	GTPCH1-deficient dopa-responsive dystonia	233910	*GCH1 (AR)*	BH4, amine replacement	primary / targeting underlying pathophysiology	IV	dx. CP; spastic diplegia	[63, 64]
	PTPS deficiency (biopterin PTS PTSP deficiency	261640	*PTS (AR)*	BH4, diet, amine replacement	primary / targeting underlying pathophysiology	IV	Dystonia; spastic extremities; generalized dystonia, choreoathetoid arm movements & axial hypotonia	[65, 66, 67]

Biochemical category	Disease name	OMIM#	Gene(s)	Treatment	Effect	Level of Evidence	CP symptoms	CP mimic reference
Neurotransmission	Sepiapterin reductase deficiency	612716	*SPR (AR)*	Amine replacement	primary / targeting underlying pathophysiology	IV	Limb spasticity, dystonic signs; ‘‘hypotonic cerebral palsy’’; dystonia, axial hypotonia; misdx. CP	[68, 69, 70]
	Succinic semialdehyde dehydrogenase deficiency (SSADH)	271980	*ALDH5A1 (AR)*	Vigabatrin	stabilizing/preventative treatment	IV	Hypotonia, ataxia; gait clumsiness, dystonia	[71, 72]
	Tyrosine hydroxylase deficiency	605407	*TH (AR)*	L-dopa substitution	primary / targeting underlying pathophysiology	IV	Spastic paraplegia /tetraparesis	[73, 74]
	Vesicular monoamine transporter 2 (VMAT2)	193001	*SLC18A2*	Dopamine aginist	primary / targeting underlying pathophysiology	IV	Dystonia	[75]
Organic acids	[illegible]-Ketothiolase deficiency	203750	*ACAT1 (AR)*	Avoid fasting, sickday management, protein restriction	stabilizing/preventative treatment	IV	Ataxia, diplegia, hypotonia	[76, 77]
	2-Methyl-3-hydroxybutyryl-CoA dehydrogenase (MHBD) deficiency	300438	*HSD17B10 (X-linked)*	Avoid fasting, sick daymanagement, isoleucine restricted diet	stabilizing/preventative treatment	IV	Ataxia, dystonia, choreoathetosis, spastic di-/tetra-plegia, hypotonia	[78]
	3-Methylcrotonyl-CoA carboxylase (MCC) deficiency	210200; 210210	*MCC1/MCC2 (AR)*	Dietary protein restriction; carnitine, glycine, biotin supplements; avoid fasting; sick day management	stabilizing/preventative treatment	IV	dx. CP	[79]
	3-Methylglutaconic aciduria type 1	250950	*AUH (AR)*	Carnitine supplements, avoid fasting, sick day management	stabilizing/preventative treatment	IV	dx. CP	[80, 81]
	Ethylmalonic encephalopathy	602473	*ETHE1 (AR)*	N-acetylcysteine, oral metronidazol	stabilizing/preventative treatment	IV	CNS malformations, episodic ataxia; pyramidal tract signs	[82, 83]

Biochemical category	Disease name	OMIM#	Gene(s)	Treatment	Effect	Level of Evidence	CP symptoms	CP mimic reference
Organic acids	Glutaric aciduria type I (GA1) aka glutaryl-CoA dehydrogenase deficiency	231670	*GCDH (AR)*	Lysine restriction, carnitine supplements	stabilizing/preventative treatment	III	Generalized spasticity, dystonia with athethosis; dx. CP; dyskinesia, dystonic tetraparesis	[84, 85, 86, 87, 88, 89]
	Isovaleric acidemia	243500	*IVD (AR)*	Dietary protein restriction, carnitine supplements, avoid fasting, sick day management	stabilizing/preventative treatment	III	Hypotonia, paresis	[90, 91]
	Multiple acyl-CoA-dehydrogenase deficiency (MADD) (aka Glutaric aciduria type II)	231680	*ETFA , ETFB, ETFDH (AR)*	Carnitine, riboflavin, [illegible]-hydroxybutyrate supplements; sick day management	primary / targeting underlying pathophysiology	IV	Encephalopathy	[92]
	Maple syrup urine disease	248600	*DBT, BCKDHB, BCKDHA (AR)*	Dietary restriction, branched amino-acids, avoid fasting, (liver transplantation)	stabilizing/preventative treatment (liver tx= primary treatment)	IV	Spastic diplegic CP ; paroxysmal dystonia	[93, 94]
	Methylmalonic acidemia (mutase deficiency)	251000	*MUT (AR)*	Dietary protein restriction, carnitine supplements, avoid fasting, sick day management	stabilizing/preventative treatment	III	Total body dystonia	[95]
	Lesch-Nyhan syndrome	300322	*HPRT1 (X-linked)*	Haematopoietic stem cell transplant	primary / targeting underlying pathophysiology	IV	dx. Athetotic/ dyskinetic CP; dystonia	[96, 97, 98, 99]
	Propionic acidemia	606054	*PCCA, PCCB (AR)*	Dietary protein restriction, carnitine supplements, avoid fasting, sick day management	primary / targeting underlying pathophysiology	III	Dystonia, hypotonia	[100, 101]
Urea cycle	Argininemia	207800	*ARG1 (AR)*	Dietary protein restriction, arginine supplement, sodium benzoate, phenylbutyrate (Liver transplantation)	stabilizing/preventative treatment (liver tx= primary treatment)	III	Spastic diplegia, ataxia, dx. CP	[102, 103, 104, 105]
	Citrullinemia, type II	605814	*SLC25A13 (AR)*	Dietary protein restriction, arginine supplement, sodium benzoate, phenylbutyrate (Liver transplantation)	stabilizing/preventative treatment (liver tx= primary treatment)	III	dx. CP; spastic quadriplegia	[106]

Biochemical category	Disease name	OMIM#	Gene(s)	Treatment	Effect	Level of Evidence	CP symptoms	CP mimic reference
Urea cycle	Ornithine transcarbamylase deficiency	311250	*OTC (X-linked)*	Dietary protein restriction, citrulline supplements, sodium benzoate/ phenylbutyrate (Liver transplantation)	stabilizing/preventative treatment (liver tx= primary treatment)	III	Hemiplegia; ataxia	[107, 108]
Vitamins/ Co-factors	Biotinidase deficiency	2532760	*BTD (AR)*	Biotin supplement	primary / targeting underlying pathophysiology	III	Spastic tetraparesis	[109]
	Biotin-thiamine-responsive basal ganglia disease	607483	SLC19A3 (AR)	Biotin supplement	primary / targeting underlying pathophysiology	IV	Ataxia, dystonia	[110, 111]
	Cerebral folate deficiency syndrome	613068	FOLR1 (AR)	Folinic acid	primary / targeting underlying pathophysiology	IV	spastic paraplegia	[112]
	Holocarboxylase synthetase deficiency	253270	HLCS (AR)	Biotin supplement	primary / targeting underlying pathophysiology	IV	dx. CP	[113]
	Molybdenum cofactor deficiency	252150	MOCS1, MOCS2, (AR)	Precursor Z/cPMP	primary / targeting underlying pathophysiology	IV	Spastic quadriplegia dx. CP	[114]
	Pyridoxamine 5'-phosphate oxidase deficiency	610090	PNPO (AR)	Pyridoxal 5'-phosphate	stabilizing/preventative treatment	IV	Spastic quadriplegia	[115, 116]

References

[30] Darras BT, Ampola MG, Dietz WH, Gilmore HE: **Intermittent dystonia in Hartnup disease**. *Pediatric Neurology* 1989, **5**(2):118-120.

[31] Salvi S, Santorelli FM, Bertini E, Boldrini R, Meli C, Donati A, Burlina AB, Rizzo C, Di Capua M, Fariello G, Dionisi-Vici C: **Clinical and molecular findings in hyperornithinemia-hyperammonemia-homocitrullinuria syndrome**. *Neurology* (2001), **57**(5):911-914.

[32] Debray FG, Lambert M, Lemieux B, Soucy JF, Drouin R, Fenyves D, Dubé J, Maranda B, Laframboise R, Mitchell, GA: **Phenotypic variability among patients with hyperornithinaemia-hyperammonaemia-homocitrullinuria syndrome homozygous for the delF188 mutation in SLC25A15**. *J Med Genet* (2008), **45**(11):759-764.

[33] Chiong MA, Procopis P, Carpenter K, Wilcken B: **Late-onset nonketotic hyperglycinemia with leukodystrophy and an unusual clinical course**. *Pediatr Neurol* (2007), **37**(4):283-286.

[34] Paine RS: **The variability in manifestations of untreated patients with phenylketonuria (phenylpyruvic aciduria)**. *Pediatrics* (1957), **20**:290-301.

[35] Pedersen HE, Birket-Smith E: **Neurological abnormalities in phenylketonuria**. *Acta Neurologica Scandinavica* (1974), **50**(5):589-598.

[36] Pineda M, Vilaseca MA, Artuch R, Santos S, García González MM, Aracil A, Van Schaftingen E, Jaeken, J: **3-phosphoglycerate dehydrogenase deficiency in a patient with West syndrome**. *Dev Med Child Neurol* (2000), **42**(9):629-633.

[37] Coşkun T, Aydin HI, Kiliç M, Dursun A, Haliloğlu G, Topaloğlu H, Karli-Oğuz K, de Koning TJ: **3-Phosphoglycerate Dehydrogenase Deficiency: a Case Report of a Treatable Cause of Seizures**. *Turk J Pediatr* (2009), **51**(6):587-592.

[38] Coman DJ, Sinclair KG, Burke CJ, Appleton DB, Pelekanos JT, O'Neil CM, Wallace GB, Bowling FG, Wang D, De Vivo DC, McGill JJ: **Seizures, ataxia, developmental delay and the general paediatrician: glucose transporter 1 deficiency syndrome**. *J Paediatr Child Health* (2006), **42**(5):263-267.

[39] Stromberger C, Bodamer OA, Stockler-Ipsiroglu S: **Clinical characteristics and diagnostic clues in inborn errors of creatine metabolism**. *J Inherit Metab Dis* (2003), **26**(2-3):299-308.

[40] Mercimek-Mahmutoglu S, Stoeckler-Ipsiroglu S, Adami A, Appleton R, Araújo HC, Duran M, Ensenauer R, Fernandez-Alvarez E, Garcia P, Grolik C, Item CB, Leuzzi V, Marguardt I, Muhl A, Saelke-Kellermann RA, Salomons GS, Schulze A, Surtees R, van der Knaap MS, Vasconcelos R, Verhoeven NM, Vilarinho L, Wilichowski E, Jakobs C: **GAMT deficiency: features, treatment, and outcome in an inborn error of creatine synthesis**. *Neurology* (2006), **67**(3):480-484.

[41] Iafolla AK, Thompson RJ, Roe CR: **Medium-chain acyl-coenzyme A dehydrogenase deficiency: clinical course in 120 affected children**. *J Pediatr* (1994), **124**(3):409-415.

[42] Bennett MJ, Gray RG, Isherwood DM, Murphy N, Pollitt RJ: **The diagnosis and biochemical investigation of a patient with a short chain fatty acid oxidation defect**. In *Inherited Disorders of Vitamins and Cofactors*. Edited by Addison GM, Barlett K, Harkness RA, Pollitt RJ. Springer Netherlands; 1985:135-136.

[43] Burlina AP, Edini C, Burlina AB: **Treatment of extrapyramidal symptoms in a patient with homozygous homocystinuria**. *J Inherit Metab Dis* (2002), **25**(2):135-136.

[44] Ekinci B, Apaydin H, Vural M, Ozekmekçi S: **Two siblings with homocystinuria presenting with dystonia and parkinsonism**. *Mov Disord* (2004), **19**(8):962-964.

[45] Thomas MA, Rosenblatt DS: **Severe methylenetetrahydrofolate reductase deficiency**. In *MTHFR Polymorphisms and Disease*. Edited by Ueland PM, Rozen R. Landes Bioscience; 2005:41-53.

[46] Willems PJ, Gatti R, Darby JK, Romeo G, Durand P, Dumon JE, O'Brien JS: **Fucosidosis revisited: a review of 77 patients**. *Am J Med Genet* (1991), **38**(1):111-131.

[47] Dusek P, Jankovic J, Le W: **Iron dysregulation in movement disorders**. *Neurobiol Dis* (2012), **46**(1):1-18.

[48] Escolar ML, Poe MD, Provenzale JM, Richards KC, Allison J, Wood S, Wenger DA, Pietryga D, Wall D, Champagne M, Morse R, Krivit W, Kurtzberg J: **Transplantation of umbilical-cord blood in babies with infantile Krabbe's disease**. *N Engl J Med* (2005), **352**(20):2069-2081.

[49] Kehrer C, Blumenstock G, Gieselmann V, Krägeloh-Mann I, German Leukonet: **The natural course of gross motor deterioration in metachromatic leukodystrophy**. *Dev Med Child Neurol* (2011), **53**(9): 850-855.

[50] Ji T, Kotagal S: **A 15-year-old with drop attacks and unsteadiness while descending stairs**. *Semin Pediatr Neurol* (2008), **15**(4), 174-177.

[51] Van Hove JLK, Lohr NJ: **Metabolic and monogenic causes of seizures in neonates and young infants**. *Mol Genet Metab* (2011), **104**(3), 214-230.

[52] Kim TJ, Kim IO, Kim WS, Cheon JE, Kwon JW, Seo JK, Yeon KM: **MR imaging of the brain in Wilson disease of childhood: Findings before and after treatment**. *AJNR Am J Neuroradiol* (2006), **27**(6):1373-1378.

[53] Fischer JC, Ruitenbeek W, Gabreels FJM, Janssen AJ, Renier WO, Sengers RCA, Stadhouders AM, ter Laak HJ, Trijbels JMF, Veerkamp JH: **A mitochondrial encephalomyopathy: The first case with an established defect at the level of coenzyme Q**. *Eur J Pediatr* (1986), **144**:441-444.

[54] Rötig A, Appelkvist E, Geromel V, Chretien D, Kadhom N, Edery P, Lebideau M, Dallner G, Munnich A, Ernster L, Rustin P: **Quinone-responsive multiple respiratory-chain dysfunction due to widespread coenzyme Q 10 deficiency**. *Lancet* (2000), **356**(9227):391-395.

[55] Fryer A, Appleton R, Sweeney MG, Rosenbloom L, Harding AE: **Mitochondrial DNA 8993 (NARP) mutation presenting with a heterogeneous phenotype including "cerebral palsy"**. *Arch Dis Child* (1994), **71**(5):419-422.

[56] Lissens W, Vreken P, Barth PG, Wijburg FA, Ruitenbeek W, Wanders RJ, Seneca S, Liebaers I, De Meirleir L: **Cerebral palsy and pyruvate dehydrogenase deficiency: identification of two new mutations in the E1 alpha gene**. *Eur J Pediatr* (1999), **158**(10):853-857.

[57] Head RA, de Goede CG, Newton RW, Walter JH, McShane MA, Brown RM, Brown GK: **Pyruvate dehydrogenase deficiency presenting as dystonia in childhood**. *Dev Med Child Neurol* (2004), **46**(10):710-712.

[58] McWilliam CA, Ridout CK, Brown RM, McWilliam RC, Tolmie J, Brown GK: **Pyruvate dehydrogenase E2 deficiency: a potentially treatable cause of episodic dystonia**. *Eur J Paediatr Neurol* (2010), **14**(4):349-353.

[59] Swoboda KJ, Saul JP, McKenna CE, Speller NB, Hyland K: **Aromatic L-amino acid decarboxylase deficiency: overview of clinical features and outcomes**. *Ann Neurol* (2003), **54**(Suppl 6):S49-S55.

[60] Lee HF, Tsai CR, Chi CS, Chang TM, Lee HJ: **Aromatic L-amino acid decarboxylase deficiency in Taiwan**. *Eur J Paediatr Neurol* (2009), **13**(2):135-140.

[61] Longo N: **Disorders of biopterin metabolism**. *J Inherit Metab Dis* (2009), **32**(3):333-342.

[62] Kurian MA, Li Y, Zhen J, Meyer E, Hai N, Christen HJ, Hoffmann GF, Jardine P, von Moers A, Mordekar SR, O'Callaghan F, Wassmer E, Wraige E, Dietrich C, Lewis T, Hyland K, Heales S Jr, Sanger T, Gissen P, Assmann BE, Reith ME, Maher ER: **Clinical and molecular characterisation of hereditary dopamine transporter deficiency syndrome: an observational cohort and experimental study**. *Lancet Neurol* (2011), **10**(1):54-62.

[63] Boyd K, Patterson V. Dopa responsive dystonia: a treatable condition misdiagnosed as cerebral palsy. Br Med J 1989;298:1019-1020.

[64] Nygaard TG, Waran SP, Levine RA, Naini AB, Chutorian AM: **Dopa-responsive dystonia simulating cerebral palsy**. *Pediatr Neurol* (1994), **11**(3):236-240.

[65] Demos MK, Waters PJ, Vallance HD, Lillquist Y, Makhseed N, Hyland K, Blau N, Connolly MB: **6-Pyruvoyl-Tetrahydropterin Synthase Deficiency With Mild Hyperphenylalaninemia**. *Ann Neurol* (2005), **58**(1):164-167.

[66] Lee NC, Cheng LY, Liu TT, Hsiao KJ, Chiu PC, Niu DM: **Long-term follow-up of Chinese patients who received delayed treatment for 6-pyruvoyl-tetrahydropterin synthase deficiency**. *Mol Genet Metab* (2006), **87**(2):128-134.

[67] Roze E, Vidailhet M, Blau N, Moller LB, Doummar D, de Villemeur TB, Roubergue A: **Long-term follow-up and adult outcome of 6-pyruvoyl-tetrahydropterin synthase deficiency**. *Mov Disord* (2006), **21**(2):263-266.

[68] Kusmierska K, Jansen EE, Jakobs C, Szymanska K, Malunowicz E, Meilei D, Thony B, Blau N, Tryfon J, Rokicki D, Pronicka E, Sykut-Cegielska J: **Sepiapterin reductase deficiency in a 2-year-old girl with incomplete response to treatment during short-term follow-up**. *J Inherit Metab Dis* (2009), **32** (Suppl 1):S5-S10.

[69] Wali GM, Thony B, Blau N: **Sepiapterin reductase deficiency: two Indian siblings with unusual clinical features**. *Mov Disord* (2010), **25**(7):954-955.

[70] Friedman J, Roze E, Abdenur JE, Chang R, Gasperini S, Saletti V, Wali GM, Eiroa H, Neville B, Felice A, Parascandalo R, Zafeiriou DI, Arrabal-Fernandez L, Dill P, Eichler FS, Echenne B, Gutierrez-Solana LG, Hoffmann GF, Hyland K, Kusmierska K, Tijssen MA, Lutz T, Mazzuca M, Penzien J, Poll-The BT, Sykut-Cegielska J, Szymanska K, Thöny B, Blau N: **Sepiapterin reductase deficiency: a treatable mimic of cerebral palsy**. *Ann Neurol* (2012), **71**(4):520-530.

[71] Gibson KM, Christensen E, Jakobs C, Fowler B, Clarke MA, Hammersen G, Raab K, Kobori J, Moosa A, Vollmer B, Rossier E, Iafolla AK, Matern D, Brouwer OF, Finkelstein J, Aksu F, Weber HP, Bakkeren JA, Gabreels FJ, Bluestone D, Barron TF, Beauvais P, Rabier D, Santos C, Lehnert W, et al: **The clinical phenotype of succinic semialdehyde dehydrogenase deficiency (4-hydroxybutyric aciduria): case reports of 23 new patients**. *Pediatrics* (1997), **99**(4):567-574.

[72] Leuzzi V, Di Sabato ML, Deodato F, Rizzo C, Boenzi S, Carducci C, Malaspina P, Liberanome C, Dionisi-Vici C: **Vigabatrin improves paroxysmal dystonia in succinic semialdehyde dehydrogenase deficiency**. *Neurology* (2007), **68**(16):1320-1321.

[73] Furukawa Y, Graf WD, Wong H, Shimadzu M, Kish SJ: **Dopa-responsive dystonia simulating spastic paraplegia due to tyrosine hydroxylase (TH) gene mutations**. *Neurology* (2001), **56**(2):260-263.

[74] Hoffmann GF, Assmann B, Bräutigam C, Dionisi-Vici C, Häussler M, de Klerk JB, Naumann M, Steenbergen-Spanjers GC, Strassburg HM, Wevers RA: **Tyrosine hydroxylase deficiency causes progressive encephalopathy and dopa-nonresponsive dystonia**. *Ann Neurol* (2003), **54**(Suppl 6):S56-65.

[75] Rilstone JJ, Alkhater RA, Minassian BA: **Brain dopamine-serotonin vesicular transport disease and its treatment**. *N Engl J Med* (2013), **368**(6):543-550.

[76] Søvik O: **Mitochondrial 2-methylacetoacetyl-CoA thiolase deficiency: an inborn error of isoleucine and ketone body metabolism**. *J Inherit Metab Dis* (1993), **16**(1):46-54.

[77] Buhaş D, Bernard G, Fukao T, Décarie JC, Chouinard S, Mitchell GA: **A treatable new cause of chorea: beta-ketothiolase deficiency**. *Mov Dis* (2013), **28**(8):1054-1056.

[78] Korman SH: **Inborn errors of isoleucine degradation: a review**. *Mol Genet Metab* (2006), **89**(4):289-299.

[79] Murayama K, Kimura M, Yamaguchi S, Shinka T, Kodama K: **Isolated 3-methylcrotonyl-CoA carboxylase deficiency in a 15-year-old girl**. *Brain Dev* (1997), **19**(4):303-305.

[80] Straussberg R, Brand N, Gadoth N: **3-Methyl glutaconic aciduria in Iraqi Jewish children may be misdiagnosed as cerebral palsy**. *Neuropediatrics* (1998), **29**(1):54-56.

[81] Pantaleoni C, D'Arrigo S, D'Incerti L, Rimoldi M, Riva D: **A case of 3-methylglutaconic aciduria misdiagnosed as cerebral palsy**. *Pediatr Neurol* (2000), **23**(5):442-444.

[82] Nowaczyk MJ, Blaser SI, Clarke JT: **Central nervous system malformations in ethylmalonic encephalopathy**. *Am J Med Genet* (1998), **75**(3):292-296.

[83] Ismail EA, Seoudi TM, Morsi EA, Ahmad AH: **Ethylmalonic encephalopathy. Another patient from Kuwait**. *Neurosciences (Riyadh, Saudi Arabia)*, **14**(1):78-80.

[84] Goodman SI, Markey SP, Moe PG, Miles BS, Teng CC: **Glutaric aciduria; a “new” disorder of amino acid metabolism**. *Biochem Med* (1975), **12**(1):12-21.

[85] Stutchfield P, Edwards MA, Gray RGF, Crawley P, Green A: **Glutaric aciduria type I misdiagnosed as Leigh's encephalopathy and cerebral palsy**. *Dev Med Child Neurol* (1985), **27**:514-521.

[86] Morton DH, Bennett MJ, Seargeant LE, Nichter CA, Kelley RI: **Glutaric aciduria type I: a common cause of episodic encephalopathy and spastic paralysis in the Amish of Lancaster County, Pennsylvania**. *Am J Med Genet* (1991), **41**:89-95.

[87] Kyllerman M, Skjeldal OH, Lundberg M, Holme I, Jellum E, von Döbeln U, Fossen A, Carlsson, G: **Dystonia and dyskinesia in glutaric aciduria type I: clinical heterogeneity and therapeutic considerations**. *Mov Dis* (1994), **9**(1):22-30.

[88] Monavari AA, Naughten ER: **Prevention of cerebral palsy in glutaric aciduria type 1 by dietary management**. *Arch Dis Child* (2000), **82**(1):67-70.

[89] Cerisola A, Campistol J, Pérez-Dueñas B, Poo P, Pineda M, García-Cazorla A, Sanmartí FX, Ribes A, Vilaseca, MA: **Seizures versus dystonia in encephalopathic crisis of glutaric aciduria type I**. *Pediatr Neuroly* (2009), **40**(6):426-431.

[90] Seashore MR: **The Organic Acidemias: An Overview**. In: *GeneReviews®* [Internet]. Edited by Pagon RA, Adam MP, Ardinger HH, et al. Seattle (WA): University of Washington, Seattle; 1993-2014 [Updated 2009 Dec 22].

[91] Albanese A, Jankovic J: *Hyperkinetic Movement Disorders: Differential Diagnosis and Treatment*. John Wiley & Sons, 2012.

[92] Olsen RKJ, Olpin SE, Andresen BS, Miedzybrodzka ZH, Pourfarzam M, Merinero B, Frerman FE, Beresford MW, Dean JC, Cornelius N, Andersen O, Oldfors A, Holme E, Gregersen N, Turnbull DM, Morris, AA: **ETFDH mutations as a major cause of riboflavin-responsive multiple acyl-CoA dehydrogenation deficiency**. *Brain* (2007), **130**(Pt 8):2045-2054.

[93] Davidson RS, Carroll NC: **Cerebral palsy associated with maple syrup urine disease**. *J Pediatr Orthop* (1982), **2**(2):165-170.

[94] Temudo T, Martins E, Poças F, Cruz R, Vilarinho L: **Maple syrup disease presenting as paroxysmal dystonia**. *Ann Neurol* (2004), **56**(5):749-750.

[95] Chakraborti S, Hasegawa H, Lumsden DE, Ali W, Kaminska M, Lin JP, Ashkan K**: Bilateral subthalamic nucleus deep brain stimulation for refractory total body dystonia secondary to metabolic autopallidotomy in a 4-year-old boy with infantile methylmalonic acidemia: case report**. *J Neurosurg Pediatr* (2013), **12**(4):374-379.

[96] Mitchell G, McInnes RR**: Differential diagnosis of cerebral palsy: Lesch-Nyhan syndrome without self-mutilation**. *Can Med Assoc J* (1984), **130**(10):1323-1324.

[97] Jinnah HA, Visser JE, Harris JC, Verdu A, Larovere L, Ceballos-Picot I, Gonzalez-Alegre P, Neychev V, Torres RJ, Dulac O, Desguerre I, Schretlen DJ, Robey KL, Barabas G, Bloem BR, Nyhan W, De Kremer R, Eddy GE, Puig JG, Reich SG, Lesch-Nyhan Disease International Study Group: **Delineation of the motor disorder of Lesch-Nyhan disease**. *Brain* (2006), **129**(Pt 5):1201-1217. doi:10.1093/brain/awl056

[98] Chiong MA, Marinaki, A, Duley J, Bennetts B, Ouvrier R, Christodoulou J: **Lesch-Nyhan disease in a 20-year- old man incorrectly described as developing “cerebral palsy” after general anaesthesia in infancy**. *J Inherit Metab Dis* (2006), **29**(4):594.

[99] Levart TK: **Rare variant of Lesch-Nyhan syndrome without self-mutilation or nephrolithiasis**. *Pediatr Nephrol* (2007), **22**(11):1975-1978.

[100] Surtees RA, Matthews EE, Leonard JV: **Neurologic outcome of propionic acidemia**. *Pediatr Neurol* (1992), **8**(5):333-337.

[101] Ozand PT, Rashed M, Gascon GG, Youssef NG, Harfi H, Rahbeeni Z, al Garawi S, al Aqeel A: **Unusual presentations of propionic acidemia**. *Brain Dev* (1994), **16 Suppl**(966):46-57.

[102] Scheuerle AE, McVie R, Beaudet AL, Shapira SK: **Arginase Deficiency Presenting as Cerebral Palsy**. *Pediatrics* (1993), **91**(5):995-996.

[103] Prasad AN, Breen JC, Ampola MG, Rosman NP: **Argininemia: a treatable genetic cause of progressive spastic diplegia simulating cerebral palsy: case reports and literature review**. *J Child Neurol* (1997), **12**(5):301-309.

[104] Lee BH, Jin HY, Kim GH, Choi JH, Yoo HW: **Argininemia presenting with progressive spastic diplegia**. *Pediatr Neurol* (2011), **44**(3):218-220.

[105] Carvalho DR, Brum JM, Speck-Martins CE, Ventura FD, Navarro MM, Coelho KE, Portugal D, Pratesi R. (2012). **Clinical features and neurologic progression of hyperargininemia**. *Pediatr Neurol* (2012), **46**(6):369-374.

[106] Issa ARA, Yadav G, Teebi AS: **Intrafamilial phenotypic variability in citrullinaemia : report of a family**. *J Inherit Metab Dis* (1988), **11**(3):306-307.

[107] Christodoulou J, Qureshi IA, McInnes RR, Clarke JT: **Ornithine transcarbamylase deficiency presenting with strokelike episodes**. *J Pediatr* (1993), **122**(3):423-425.

[108] Saudubray JM, Desguerre I, Sedel F, Charpentier C: **Acute symptoms in the neonatal period and early infancy**. In *A clinical approach to inherited metabolic diseases*. Springer Berlin Heidelberg; 2006:6-10.

[109] Komur M, Okuyaz C, Ezgu F, Atici A: **A girl with spastic tetraparesis associated with biotinidase deficiency**. *Eur J Paediatr Neurol* (2011), **15**(6): 551-553.

[110] Ozand PT, Gascon GG, Al Essa M, Joshi S, Al Jishi E, Bakheet S, Al Watban J, Al-Kawi MZ, Dabbagh O: **Biotin-responsive basal ganglia disease: a novel entity**. *Brain* (1998), **121**(Pt 7):1267-1279.

[111] Alfadhel M, Almuntashri M, Jadah RH, Bashiri FA, Al Rifai MT, Al Shalaan H, Al Balwi M, Al Rumayan A, Eyaid W, Al-Twaijri W: **Biotin-responsive basal ganglia disease should be renamed biotin-thiamine-responsive basal ganglia disease: a retrospective review of the clinical, radiological and molecular findings of 18 new cases**. *Orphanet J Rare Dis* (2013), **8**(1):83.

[112] Hansen FJ, Blau N: **Cerebral folate deficiency: life-changing supplementation with folinic acid**. *Mol Genet Metab* (2005), **84**(4):371-373.

[113] Livne M, Gibson KM, Amir N, Eshel G, Elpeleg ON: **Holocarboxylase Synthetase Deficiency: A Treatable Metabolic Disorder Masquerading as Cerebral Palsy**. *J Child Neurol* (1994), **9**(2):170-172.

[114] Kikuchi K, Hamano S, Mochizuki H, Ichida K, Ida H: **Molybdenum cofactor deficiency mimics cerebral palsy: differentiating factors for diagnosis**. *Pediatr Neurol* (2012), **47**(2):147-149.

[115] Mills PB, Surtees RAH, Champion MP, Beesley CE, Dalton N, Scambler PJ, Heales SJR., Briddon A, Scheimberg I, Hoffmann GF, Zschocke J, Clayton PT: **Neonatal epileptic encephalopathy caused by mutations in the PNPO gene encoding pyridox(am)ine 5-prime-phosphate oxidase**. *Hum Molec Genet* (2005), **14**:1077-1086.

[116] Mills PB, Camuzeaux SS, Footitt EJ, Mills KA, Gissen P, Fisher L, Das KB, Varadkar SM, Zuberi S, McWilliam R, Stödberg T, Plecko B, Baumgartner MR, Maier O, Calvert S, Riney K, Wolf NI, Livingston JH, Bala P, Morel CF, Feillet F, Raimondi F, Del Giudice E, Chong WK, Pitt M, Clayton PT: **Epilepsy due to PNPO mutations: genotype, environment and treatment affect presentation and outcome**. *Brain* (2014), **137**(Pt 5):1350-1360.
